# Reliability and Validity of a New Transfer Assessment Form for Stroke Patients

**DOI:** 10.1002/pmrj.12400

**Published:** 2020-06-10

**Authors:** Shin Kitamura, Yohei Otaka, Yudai Murayama, Kazuki Ushizawa, Yuya Narita, Naho Nakatsukasa, Kunitsugu Kondo, Sachiko Sakata

**Affiliations:** ^1^ Department of Rehabilitation Medicine Tokyo Bay Rehabilitation Hospital Chiba Japan; ^2^ Department of Rehabilitation Medicine I, School of Medicine Fujita Health University Aichi Japan

## Abstract

**Introduction:**

Transferring is a basic skill that is essential for mobility independence and indispensable for expanding activities of daily living of stroke patients using a wheelchair. Therefore, transfer independence is an important issue that greatly affects daily life in the hospital and at home. To offer an effective intervention to acquire a skill, developing an assessment for individual subtasks that comprise transferring would assist the identification of specific tasks that prevent independence in patients and facilitate interventions to improve transferring independence.

**Objective:**

To examine the reliability and validity of a newly developed transfer assessment form, the Bed‐wheelchair transfer Tasks Assessment Form (BTAF), for stroke patients to evaluate subtasks required for transferring.

**Design:**

Validation and test‐retest studies.

**Setting:**

Subacute rehabilitation wards in Japan.

**Participants:**

A total of 82 therapists for verifying content validity; 30 patients for validation and test‐retest study.

**Interventions:**

Not applicable.

**Main Outcome Measures:**

The content validity was initially assessed based on a questionnaire. Subsequently, four occupational therapists used the form to evaluate the video‐recorded transferring performances of stroke participants. Two assessors evaluated each performance once and then 2 weeks later. The inter‐rater reliability, intra‐rater reliability, internal consistency, and concurrent validity were examined.

**Results:**

Fleiss's κ coefficient for inter‐rater reliability for each item of the form was 0.66 or more. Cohen's κ coefficient for intra‐rater reliability for each item was 0.73 or more. Cronbach's coefficient alpha ranged from 0.90 to 0.93. Spearman's rank correlation coefficients between the mean scores of our form and scores of the functional independence measure item “transfer to bed/chair/wheelchair” ranged from 0.53 to 0.78 (*P* < .01).

**Conclusions:**

The form demonstrated good reliability and validity. Its usefulness and efficacy should be further investigated in stroke patients to facilitate rehabilitation.

## Introduction

Among activities of daily living (ADL), transferring is a basic skill that is essential for mobility independence and indispensable for expanding ADLs of stroke patients using a wheelchair. However, transferring involves a high risk of falling^1^ and often requires observation or assistance by others. Therefore, transfer independence in stroke patients is an important issue that greatly affects daily life in the hospital and at home.

To offer an effective intervention to acquire a skill, it is necessary to evaluate the skill appropriately and clarify its problems. To assess transferring, the transfer items of the functional independence measure (FIM)^2^ and Barthel Index^3^ are frequently used.^4^ Although these established instruments can evaluate the degree of independence of movement as a whole, details of its components, such as which point of transfer requires assistance, cannot be assessed. However, the transfer assessment instrument (TAI)^5^, ^6^ can obtain details of the degree of independence by subdividing each process of transfer. Unfortunately, the TAI assesses only the skills involved in transferring and does not include tasks related to the transfer, such as wearing shoes and tasks performed while sitting on a bed. A previous study has suggested that assessment of the individual components comprising each daily activity is effective for determining rehabilitation goals and treatment planning.^7^ This type of assessment tool has been developed for dressing. A detailed assessment of component tasks of upper‐body dressing can document the most difficult components involved in dressing and assess the motor skills required for dressing independence.^8^

Furthermore, to become fully independent during transferring in real situations, it is necessary to perform all tasks related to transferring independently. Aside from the main task of transferring from a bed to a wheelchair, skills involving taking off a bed comforter, wearing shoes, placing the wheelchair in the proper position at the bedside, and others, must be acquired for true independence during transferring.^9^ When planning the intervention to achieve the patient's transferring independence, clinicians should assess a whole series of subtasks, including its associated tasks. However, to the best of our knowledge, there are no assessment tools for evaluating a series of transferring subtasks, including pre‐transferring and post‐transferring tasks.

Assessments of the individual subtasks that comprise transferring can help us identify specific problems and facilitate interventions to accomplish the individual's independence. This study aimed to verify the reliability and validity of a new transfer assessment tool, the Bed‐wheelchair transfer Tasks Assessment Form (BTAF), which we developed to evaluate various subtasks that comprise transferring.

## Methods

### *Study Setting and Outline*

The study was conducted at a 160‐bed subacute rehabilitation hospital in Japan where medical insurance paid for the rehabilitation of stroke patients who were admitted to the wards within 2 months of onset and up to 6 months after hospitalization.^10^ At our hospital, we developed the BTAF to evaluate the series of tasks comprising transferring based on expert input; the BTAF has been used for the past 10 years. It was developed from the need to assess what was necessary for the independence of toileting for planning an individualized approach and also to make an appropriate judge for the independence of the task in actual clinical settings. In the present study, we examined the content validity, inter‐rater reliability, intra‐rater reliability, and concurrent validity of the BTAF. The protocol for this study was approved by the appropriate ethics committee.

### *Assessment Form*

BTAF is a tool used to evaluate the bed‐wheelchair transferring activities of patients who have experienced a paretic stroke. The BTAF classifies the series of transferring subtasks into 25 items. Each subtask is judged as follows: A, independent (the participant can complete the task without requiring intervention by the therapist); B, requires supervision or verbal assistance (the participant can complete a task with supervision or needs verbal assistance by the therapist); C, requires assistance (the therapist needs to physically assist the participant or manipulate equipment to complete the task); and N, not applicable (the participant does not need to perform the task; for example, the task of “put the foot on the footrest” applies only to those with wheelchairs with a footrest). The form has been designed to evaluate mainly standing pivot transferring, which is the most common type of transferring for patients with hemiparesis. However, we can use the form for various types of transfers, such as nonstanding transferring by scoring the items not required for the transferring as N.

Because the purpose of performing the evaluation using the form is to determine the specific component that requires intervention in a real‐world environment, all subtasks performed during transferring were sequentially included as they appeared during the real situation. Therefore, some items were duplicated. Subtasks such as “press the nurse call button,” “keep sitting on the bedside,” and “turn while standing” are required twice during one single transferring activity. Although the task name is the same, the situation and environment are different. For example, the situation of the first “press the nurse call button” requires the patient to press the nurse call button to call a staff member to help in transferring to the wheelchair from the bed before getting up; the second one requires the patient to call the staff member for help in transferring to the bed from the wheelchair before standing up from sitting in the wheelchair. The position of the button is different for these two situations, and the patient's posture is also different. This sequential, step‐by‐step evaluation of all subtasks during transferring is one of the distinct characteristics of the form.

### *Content Validity of the BTAF*

The content validity of the BTAF was performed based on the Delphi method.^11^ We used a questionnaire to survey the appropriateness of the BTAF. Each item of the BTAF was judged based on whether the assessment was able to (1) clarify specific steps (or subtasks) that prevent independent transferring, (2) help plan a better rehabilitation program, and (3) be used as a criterion for independence. The responders of the questionnaire were 82 therapists who worked at the hospital. Among them, 38 were occupational therapists (experience, 1‐28 years; median, 3 years), and 44 were physical therapists (experience, 1‐19 years; median, 3 years). These occupational therapists had used the form in a clinical setting. These physical therapists, however, had never used the form before the study. The appropriateness of each item of the BTAF was judged using a 5‐point Likert scale as follows: 5, very necessary; 4, mostly necessary; 3, neither necessary nor unnecessary; 2, almost not necessary; and 1, not necessary at all. In addition, we asked all responders to mention reasons for all items that were scored as 3 or less. The form was revised based on the results of this questionnaire. A given item was included if ≥80% of the participants scored it as 4 or 5. All other items were either revised or deleted. A second questionnaire‐based survey was conducted to assess the appropriateness of the corrected form. During this survey, participants were allowed to access the overall scores of the first survey for each item. Based on the results of the two surveys, three experts (two occupational therapists and one physiatrist) examined the suitability of each item and finalized the content of the form.

### *Inter‐Rater and Intra‐Rater Reliability and Concurrent Validity*

We enrolled 30 patients who were admitted to the hospital from June 2016 to June 2018 after a stroke. Participants who were using a wheelchair and who used standing pivot transferring between the bed and the wheelchair were included. All participants were recruited by convenience sampling and provided written informed consent before they participated in the study. Background information of the participants, including the motor items of the Stroke Impairment Assessment Set (SIAS)^12^, ^13^ and the FIM, are shown in Table 1. The motor items of the SIAS consist of the upper limb (two items) and lower limbs (three items) on the hemiparetic side, with six grades ranging from 0 (total paralysis) to 5 (normal) for each item. The FIM score evaluated by a nurse within 1 week before or after the TTAF evaluation was adopted. As shown in Table 1, the participants had a wide range of severity for paresis and ADLs.

**Table 1:** Participant characteristics (n = 30)

Sex, male/female	17/13
Age, y	72.7 ± 9.9
Type of stroke, hemorrhage/infarction	14/16
Side of hemiparesis, right/left/bilateral	14/13/3
Days after stroke onset	85.5 ± 39.2
Days after admission to rehabilitation hospital	49.1 ± 35.6
Stroke impairment assessment set: Motor function
Knee‐mouth	1.5 (0–5)
Finger‐function	1 (0–5)
Hip‐flexion	2 (0–5)
Knee‐extension	2 (0–5)
Foot‐pat	1 (0–5)
Functional independence measure
Motor score	40 (13‐59)
Cognitive score	22.5 (9‐35)
Total score	62 (22‐92)

Values are presented as numbers, mean ± SD, or median (minimum‐maximum).

For participants with bilateral paresis, the scores for the worse side were adopted for the motor function of the stroke impairment assessment set.

Participants were asked to perform a series of transferring tasks from sitting in the wheelchair until lying down on the bed and vice versa in the hospital room. The video‐recorded performances were assessed using the BTAF by four occupational therapists with 2, 3, 4, and 6 years of clinical experience. A numerical scoring system for the BTAF was provided as follows: A, 3 points; B, 2 points; and C, 1 point. We used the individual score of each item as well as the mean score of the form for the analyses of the inter‐rater reliability, intra‐rater reliability, internal consistency, and concurrent validity. The mean score was calculated by dividing the total score by the number of items (excluding the items marked as not applicable [N]).

### *Inter‐Rater Reliability*

Fleiss's κ coefficient was calculated by using the assessment scores for each item, as marked by the four assessors. The inter‐rater reliability between the mean score given by each assessor was also examined by using intra‐class correlation coefficients (ICCs: [2, 1]).

### *Intra‐Rater Reliability*

The assessment was repeated following a 2‐week interval by two assessors (with 2 and 6 years of clinical experience) who had also performed the first assessment. The agreement between the two assessments of each therapist was evaluated using Cohen's κ coefficient. The intra‐rater reliability of the mean score was also examined by using ICCs (1, 1). At the mean score, the minimal detectable change with a confidence level of 95% (MDC_95_), the threshold for determining clinical changes beyond measurement error, was also calculated based on the standard error of measurement (SEM) of the intra‐rater reliability.^14^

### *Internal Consistency*

Cronbach's coefficient alpha for each assessor was calculated based on the item scores of all participants.

### *Concurrent Validity*

Four assessors who scored the BTAF also scored the transferring item of the FIM by watching the same video‐recorded performance. The correlation analyses between the mean scores of the BTAF and the scores of the FIM item “transfer to bed/chair/wheelchair” were performed for each assessor using Spearman's rank correlation coefficients. In addition, to examine whether the BTAF could identify newer aspects of the transferring performance compared with the FIM, we compared the score of each BTAF item among participants who had the same scores on the FIM item “transfer to bed/chair/wheelchair.” The median of the BTAF scores and the mode of the FIM scores among these four assessors were adopted for this analysis. When calculating this median BTAF score, the “N” was excluded from the calculation if one or two among four assessors judged N for the item, and the “N” was adopted if more than two assessors judged N for the item.

All statistical analyses were performed with the R package (R version 3.3.2). *P* < .05 was considered statistically significant. Reliability coefficients were interpreted as follows: 0‐0.20, slight; 0.21‐0.40, fair; 0.41‐0.60, moderate; 0.61‐0.80, substantial; and 0.81‐1.00, almost perfect agreement.^15^

## Results

### *Content Validity*

**Figure 1:**
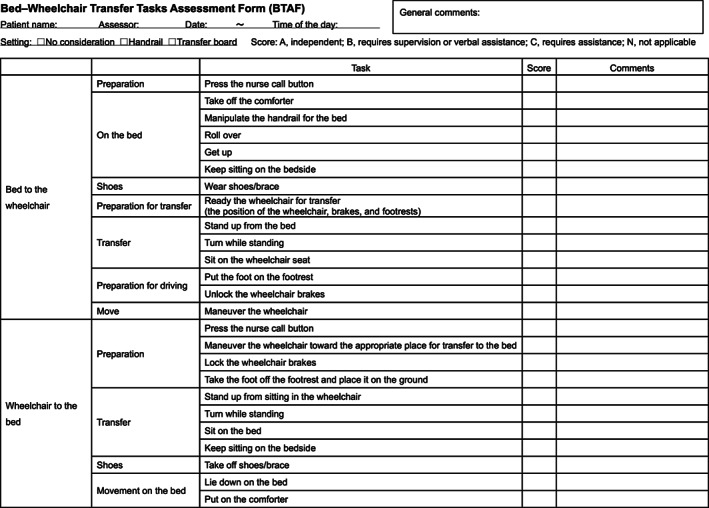
Bed‐wheelchair transfer tasks assessment form (BTAF).

The first questionnaire was sent to 82 therapists, of which 71 responded (collection rate, 87%). Among these 71 responses, 64 were valid (78%; physical therapists, 34; occupational therapists, 29; unknown, 1). Twenty‐four items of the BTAF satisfied the consensus criteria (≥80% of the participants scored 4 or 5 for each item), but the item “manipulate the handrail for the bed” did not satisfy the criteria (percentage of content was 55%). With regard to the necessity of that item, reasons for answering “3: neither” to “1: not necessary at all” were as follows: “some people do not need to use handrails” and “the handrail of the bed fence should be fixed in a correct position beforehand.” Almost all descriptions were about the low frequency of handrail use. There were few answers to refer to the low necessity of the item. According to a previous study,^9^ this item was regarded as the transfer skill item that was most difficult for patients. The experts concluded that it was an important item for patients who need to use the handrail of the bed fence, although the frequency of this task is relatively low. Therefore, we decided to retain the item. The second questionnaire was sent to 82 people, of whom 67 responded (collection rate, 82%). All responses were valid (82%; physical therapists, 32; occupational therapists, 30; unknown, 5). As with the first survey, the second survey revealed that all items except for “manipulate the handrail for the bed (58%)” satisfied the content criteria, indicating high content validity. After another discussion among the experts, we decided to include “manipulate the handrail for the bed” as an item and adopted all 25 items for the final assessment form (Figure 1). There was no apparent difference in the percentages of participants who agreed for each item between occupational therapists who have the clinical experience of using BTAF and physical therapists with no experience; the average percentages of participants who agreed for the items among occupational therapists and physical therapists was 95.7% and 92.9% in the first survey and 95.6% and 96.2% in the second survey, respectively.

### *Inter‐Rater and Intra‐Rater Reliability and Concurrent Validity*

Participant characteristics, including the FIM and SIAS, are presented in Table 1.

### *Inter‐Rater Reliability*

The minimum Fleiss's κ coefficient value was 0.66, indicating at least substantial inter‐rater reliability for each item of the BTAF (Table 2). The inter‐rater reliability of the mean score was almost perfect, with an ICC of 0.98 (95% confidence interval [CI], 0.96‐0.99).

**Table 2:** Reliability of each item of the bed‐wheelchair transfer tasks assessment form (BTAF) (n = 30)

Item	Inter‐rater (Fleiss's κ)	Intra‐rater (Cohen's κ)	
	Assessors 1–4	Assessor 1	Assessor 2
1. Press the nurse call button	0.84	0.78	1.00
2. Take off the comforter	0.84	0.88	0.94
3. Manipulate the handrail for the bed	0.80	0.78	0.86
4. Roll over	0.76	0.90	0.93
5. Get up	0.81	0.80	1.00
6. Keep sitting on the bedside	0.67	0.79	0.73
7. Wear shoes/brace	0.86	1.00	0.86
8. Ready the wheelchair for transfer (position of the wheelchair, brakes, and footrests)	0.74	0.87	0.82
9. Stand up from the bed	0.67	0.77	0.83
10. Turn while standing	0.80	0.84	0.90
11. Sit on the wheelchair seat	0.77	0.73	0.83
12. Put the foot on the footrest	0.88	0.91	0.95
13. Unlock the wheelchair brakes	0.76	0.77	0.87
14. Maneuver the wheelchair	0.69	0.92	0.74
15. Press the nurse call button	0.82	0.93	0.94
16. Maneuver the wheelchair toward the appropriate place for transfer to the bed	0.90	0.86	0.81
17. Lock the wheelchair brakes	0.83	0.94	1.00
18. Take the foot off the footrest and place it on the ground	0.87	0.95	0.90
19. Stand up from sitting in the wheelchair	0.70	0.89	0.95
20. Turn while standing	0.67	0.79	0.94
21. Sit on the bed	0.67	0.79	0.89
22. Keep sitting on the bedside	0.66	0.89	0.80
23. Take off shoes/brace	0.76	0.87	0.88
24. Lie down on the bed	0.82	1.00	0.93
25. Put on the comforter	0.83	0.94	1.00

The items are listed in the order of their performance.

### *Intra‐Rater Reliability*

The minimum Cohen's κ coefficient value for each assessor was 0.73, indicating at least substantial intra‐rater reliability for each BTAF item (Table 2). The ICC values were calculated as 0.99 (95% CI, 0.97‐0.99) and 0.99 (95% CI, 0.97‐0.99) by the two assessors, indicating almost perfect inter‐rater reliability of the mean BTAF score. The values of SEM and MDC by the two assessors were the same, at 0.07 and 0.21 points, respectively. These values indicate that if a change of ≥0.22 points is observed when evaluating using the BTAF twice for the same subject, a “true change” is considered occurring in the subject.

### *Internal Consistency*

The Cronbach's coefficient alpha values for the four assessors were 0.93, 0.93, 0.90, and 0.92, indicating almost perfect internal consistency.

### *Concurrent Validity*

**Figure 2:**
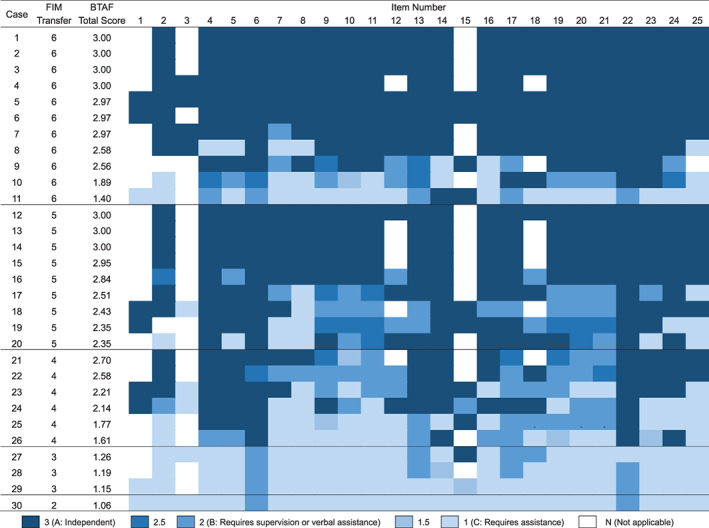
Score of each item of the bed‐wheelchair transfer tasks assessment form (BTAF) and the scores of the functional independence measure (FIM). The item numbers correspond to the items listed in Table 2. Color‐coding represents the median score provided for each item of the BTAF. BTAF total score presented is the mean of four assessors.

Spearman's rank correlation coefficients between the mean BTAF scores and the scores on the FIM item “transfer to bed/chair/wheelchair” for the four assessors were 0.68, 0.78, 0.53, and 0.61 (*P* < .01). The scores of each BTAF item and the scores for the FIM item “transfer to bed/chair/wheelchair” for individual participants are shown in Figure 2. The results demonstrated that participants who had the same FIM scores had different scores for the various BTAF items as well as different total scores.

## Discussion

The BTAF, a new transfer assessment tool, was found to have good content validity, inter‐rater reliability, intra‐rater reliability, internal consistency, and concurrent validity. Because the transfer assessment form verified in this study has been used at our hospital, the occupational therapists, but not the physical therapist, who participated in the questionnaire, had used the assessment form in a clinical setting. Therefore, the answers for the appropriateness of each item of the assessment form were at risk of bias. In other words, the answers were based on the clinical experience of using the form; and the form was already considered highly valid in the current clinical setting. In addition, BTAF was also validated for the population who had never used it (ie, physical therapists).

The inter‐rater reliability, intra‐rater reliability, internal consistency, and concurrent validity were relatively good. Among these, Fleiss's κ coefficients of inter‐rater reliability for six items, “keep sitting on the bedside (after getting up),” “stand up from the bed,” “maneuver the wheelchair,” “turn while standing (wheelchair to bed),” “sit on the bed,” and “keep sitting on the bedside (after sitting on the bed),” were between 0.66 and 0.69, thereby showing relatively low coefficients among the items. There are a few possible reasons for the relatively low agreement for these items. As regards the items “keep sitting on the bedside,” it might have been difficult for the rater to judge between the scores of A (independent) and B (requires supervision or verbal assistance) for the task. The assessors were required to score participants by watching video recordings of their performance only once. It may be difficult to fully appreciate a given participant's stability while performing the task, especially those that are performed in the sitting position. In the item “maneuver the wheelchair,” the assistant sometimes touched the handgrip. However, it is difficult to judge from the video whether the assistant was helping to push the wheelchair or just touching the handgrip. The other three items, “stand up from the bed,” “turn while standing (wheelchair to bed),” and “sit on the bed,” were related to a series of movement tasks between the wheelchair and the bed. It seemed that actual movements for these items had some variability. For example, some participants transferred to the bed by laterally moving without standing upright or turning the body fully. In these cases, it could be difficult to judge whether the behavior was independent. Furthermore, the position of the camera could not be the same among the participants because the task was performed in each participant's room. In addition, only one camera was used. Therefore, it may have been difficult to judge the tasks correctly by only using the video, especially on behavior that included turning the body; therefore, it is possible that this underestimated the reliability. Although these items had relatively low reliability, it was within the acceptable range.

The minimum Spearman's rank correlation coefficient between the mean BTAF scores and the score on the FIM item “transfer to bed/chair/wheelchair” calculated for the four assessors was 0.53, indicating a relatively low correlation. Because BTAF enables more detailed evaluations, evaluation tasks in common with FIM are only a part of the whole. This may be the reason that the correlation with FIM was not so high. Actually, participants who had the same scores on the FIM item “transfer to bed/chair/wheelchair” were found to have different scores for other BTAF items, indicating the ability of BTAF to provide a more detailed evaluation of transferring performance. The FIM score does not identify the specific actions that require assistance. However, the BTAF indicates the degree of independence for individual subtasks comprising transferring. Therefore, the BTAF may be more suitable in the clinical setting to identify specific areas of weaknesses that require intervention.

This study had some limitations. A few items such as “press the nurse call button” were judged as N in many cases, and therefore, the reliability could not be sufficiently examined for these items. This might be caused by the study design, where the task was simulated instead of actually performed. Further verification is required for these items. In addition, the present study was conducted in a single facility where the assessment tool was developed. Future studies are needed to explore whether similar results can be obtained when the assessment form is used at other facilities with different environments or community settings such as at the patient's home.

## Conclusions

This study demonstrated that the BTAF, our newly developed transfer assessment form, had relatively high reliability and validity. Future studies should explore the usefulness and efficacy of the BTAF for stroke patients to facilitate their rehabilitation.

## References

[1] Nyberg L , Gustafson Y . Patient falls in stroke rehabilitation. A challenge to rehabilitation strategies. Stroke. 1995;26:838‐842. 7740577 10.1161/01.str.26.5.838

[2] Data Management Service of the Uniform Data System for Medical Rehabilitation and the Center for Functional Assessment Research . Guide for use of the uniform data set for medical rehabilitation including the functional independence measure (FIM), Version 3.0. Buffalo, NY: State University of New York; 1990.

[3] Mahoney FI , Barthel DW . Functional evaluation: the Barthel index. Md State Med J. 1965;14:56‐61. 14258950

[4] Hsieh CL , Hoffmann T , Gustafsson L , Lee YC . The diverse constructs use of activities of daily living measures in stroke randomized controlled trials in the years 2005‐2009. J Rehabil Med. 2012;44:720‐726. 22772830 10.2340/16501977-1008

[5] McClure LA , Boninger ML , Ozawa H , Koontz A . Reliability and validity analysis of the transfer assessment instrument. Arch Phys Med Rehabil. 2011;92:499‐508. 21276957 10.1016/j.apmr.2010.07.231

[6] Tsai CY , Rice LA , Hoelmer C , Boninger ML , Koontz AM . Basic psychometric properties of the transfer assessment instrument. Arch Phys Med Rehabil. 2013;94:2456‐2464. 23685096 10.1016/j.apmr.2013.05.001

[7] Klein RM , Bell B . Self‐care skills: behavioral measurement with Klein‐Bell ADL scale. Arch Phys Med Rehabil. 1982;63:335‐338. 7092535

[8] Suzuki M , Yamada S , Omori M , et al. Development of the upper‐body dressing scale for a buttoned shirt: a preliminary correlational study. Am J Phys Med Rehabil. 2008;87:740‐749. 18716486 10.1097/PHM.0b013e31818378b0

[9] Sakata S , Otaka Y , Sato M , Sakagami S , Kondo K . The difficult components of a transfer task in post‐stroke hemiplegia. Sogo Rihabiriteshon. 2014;42:763‐770.

[10] Miyai I , Sonoda S , Nagai S , et al. Results of new policies for inpatient rehabilitation coverage in Japan. Neurorehabil Neural Repair. 2011;25:540‐547. 21451116 10.1177/1545968311402696

[11] Jones J , Hunter D . Consensus methods for medical and health services research. BMJ. 1995;311:376‐380. 7640549 10.1136/bmj.311.7001.376 PMC2550437

[12] Chino N , Sonoda S , Domen K , Saitoh E , Kimura A . Stroke impairment assessment set (SIAS): a new evaluation instrument for stroke patients. Jpn J Rehabil Med. 1994;31:119‐125.

[13] Chino N , Sonoda S , Domen K , Saitoh E , Kimura A . Stroke impairment assessment set (SIAS). In: Chino N , Melvin JL , eds. Functional Evaluation of Stroke Patients. Tokyo: Springer‐Verlag Tokyo; 1996:19‐31.

[14] Faber MJ , Bosscher RJ , van Wieringen PC . Clinimetric properties of the performance‐oriented mobility assessment. Phys Ther. 2006;86:944‐954. 16813475

[15] Landis JR , Koch GG . The measurement of observer agreement for categorical data. Biometrics. 1977;33:159‐174. 843571

